# *Orthotospovirus tomatomaculae* (Tomato spotted wilt virus [TSWV]) infects ovules and pollen to achieve vertical transmission in *Capsicum annuum*

**DOI:** 10.1128/jvi.00223-26

**Published:** 2026-06-12

**Authors:** Fan Zhang, Mao-Sen Wang, Xiao-Xia Su, Akram Salah, Kuan-Yu Zheng, Zhong-Kai Zhang

**Affiliations:** 1 Yunnan Academy of Agricultural Sciences/Key Lab of Southwestern Crop Gene Resources and Germplasm Innovation Ministry of Agriculture and Rural Affairs/ Key Lab of Agricultural Biotechnology of Yunnan Province/ Yunnan Seed Laboratory, Biotechnology and Genetic Germplasm Resources Research Institute, Kunming, Yunnan, China; 2 School of Agriculture, Yunnan University 12635, Kunming, Yunnan, China; 3 Department of Agronomy, Faculty of Agriculture, Lasbela University of Agriculture, Water & Marine Sciences 194854 , Uthal, Balochistan, Pakistan; Tsinghua University, Beijing, China

**Keywords:** TSWV, seed transmission, pollen, ovule, cross-pollination, *Capsicum annuum*, virus epidemiology

## Abstract

*Orthotospovirus tomatomaculae* (Tomato spotted wilt virus, TSWV) is an economically important plant pathogen reported to be transmissible by seeds. However, the pathways by which the virus enters the seed remain poorly understood. Here, we characterized the distribution and vertical transmission pathways of TSWV within reproductive organs of *Capsicum annuum*. Transmission electron microscopy (TEM) confirmed systemic distribution of TSWV throughout floral tissues, with virions detected in anthers, pollen grains, ovaries, ovules, and styles. Immunofluorescence histochemistry further revealed accumulation of the TSWV nucleocapsid (N) protein in the reproductive organs, with the strongest signals in anther septa, vascular bundles, and ovule integuments. Using reverse transcription-quantitative PCR (RT-qPCR) with an absolute standard curve, we detected the highest TSWV accumulation in anthers, ovaries, and styles, and lower levels in pollen grains. Cross-pollination assays demonstrated seed transmission via pollen (40%) and infected maternal tissue/ovules (30%), while self-pollination of infected plants resulted in 96% infected seeds. Testing of progeny seedlings showed infection rates of 20% (pollen-mediated), 30% (ovule-mediated), and 90% (self-pollination of infected parents). Together, these data support vertical transmission of TSWV through two reproductive routes, pollen and ovule, associated with providing a mechanistic framework for mitigating seed-borne spread in chili pepper.

**IMPORTANCE:**

Tomato spotted wilt virus (TSWV) causes substantial yield losses worldwide, yet the mechanisms underlying its seed transmission remain insufficiently defined. We provide anatomical and molecular evidence that TSWV colonizes key reproductive tissues of *Capsicum annuum*, including anthers, pollen grains, ovaries, and ovules. Using controlled cross-pollination, we demonstrate that vertical transmission can occur through infected pollen (40% seed infection; 20% seedling infection) and through infected maternal tissues/ovules (30% seed infection; 30% seedling infection). Mechanistically, these data reveal two independent routes for vertical transmission: pollen-borne and ovule-borne. This finding directly supports two evidence-based management interventions: (i) the removal of infected male plants from seed production fields to block pollen-borne spread, and (ii) the selection of virus-free maternal lines to minimize ovule-mediated transmission. Incorporating both pollen- and ovule-associated transmission into epidemiological models and resistance breeding programs will therefore be critical for mitigating seed-borne TSWV losses in chili pepper.

## INTRODUCTION

Plant viruses employ diverse transmission strategies, with seed transmission representing a critical route that enables viral persistence across plant generations (1). To date, at least 231 plant viruses and viroids have been confirmed as seed transmissible (1–3). Seed-transmitted viruses pose a substantial global agricultural threat, as they facilitate long-distance dissemination through international seed trade and serve as critical primary inoculum sources that drive secondary transmission cycles and epidemic outbreaks in field settings (4–6).

*Orthotospovirus tomatomaculae* (formerly known as Tomato spotted wilt virus [TSWV]), a member of the genus *Orthotospovirus* (family *Tospoviridae*), is one of the most economically and scientifically important plant viruses worldwide (7–10). The TSWV virions are spherical, membrane-enveloped particles with a diameter traditionally reported as 80 to 120 nm (11). However, recent cryo-electron microscopy has revealed a broader size distribution, ranging from 65 to 135 nm (12). The virus possesses a tripartite negative-sense/ambisense RNA genome consisting of large (L), medium (M), and small (S) segments (11). The L segment encodes the RNA-dependent RNA polymerase (RdRp), which catalyzes viral genome replication and transcription (10, 13, 14). The M segment encodes a movement protein (NSm) that facilitates cell-to-cell spread, as well as two glycoproteins (Gn and Gc) that are essential for host entry and thrips-mediated transmission (15, 16). The S RNA encodes the nucleocapsid protein (N) and the silencing suppressor (NSs), the latter of which enhances viral pathogenicity (13, 17).

TSWV is primarily transmitted in a persistent-propagative manner by *Frankliniella occidentalis* (western flower thrips [WFT]) (18, 19). However, vertical transmission through seeds has been reported. In pepper, TSWV was detected in the endosperm but not the embryo and was also detected in the second generation of newly germinated seedlings (20). Similar observations have been made for another orthotospovirus, soybean vein necrosis virus, in soybean (21). However, conflicting results exist in tomato: some studies detected TSWV in tomato seeds (22, 23), whereas others failed to detect it (24, 25). Despite these reports, the disparate and often incomplete findings underscore that seed transmission of TSWV remains an understudied area, and the precise mechanism by which TSWV enters and persists in developing seeds remains unclear.

For seed-borne viruses, two non-mutually exclusive pathways have been proposed to contribute to seed transmission. First pollen-associated transmission may occur when the virus present in developing or mature pollen grains has access to the ovules during fertilization, enabling infection of seed tissues (4, 26). The second pathway involves direct infection of the ovules, occurring pre-fertilization or post-fertilization, thereby establishing the virus in the developing embryo (6, 27). Both routes have been documented in diverse seed-borne plant virus systems.

Although previous studies have demonstrated that TSWV can be transmitted to seeds in pepper (20), the seed transmission pathway of TSWV in pepper remains poorly understood. Therefore, to clarify this pathway, we employed transmission electron microscopy (TEM) and immunofluorescence histochemistry to examine viral localization and reverse transcription-quantitative PCR (RT-qPCR) to quantify viral RNA accumulation in *Capsicum annuum* reproductive tissues. We confirmed the presence of TSWV in key reproductive tissues, including anthers, pollen grains, ovaries, and ovules. Through controlled cross-pollination experiments, we demonstrated that TSWV can be transmitted to the next generation via both pollen and ovules. Together, these findings establish a mechanistic framework for understanding seed-borne TSWV, directly supporting management strategies such as monitoring infected male and female parent plants and implementing pollen-targeted seed health assays to reduce crop losses in chili pepper production.

## RESULTS

### Systemic infection of TSWV in *C. annuum*

**Fig 1:**
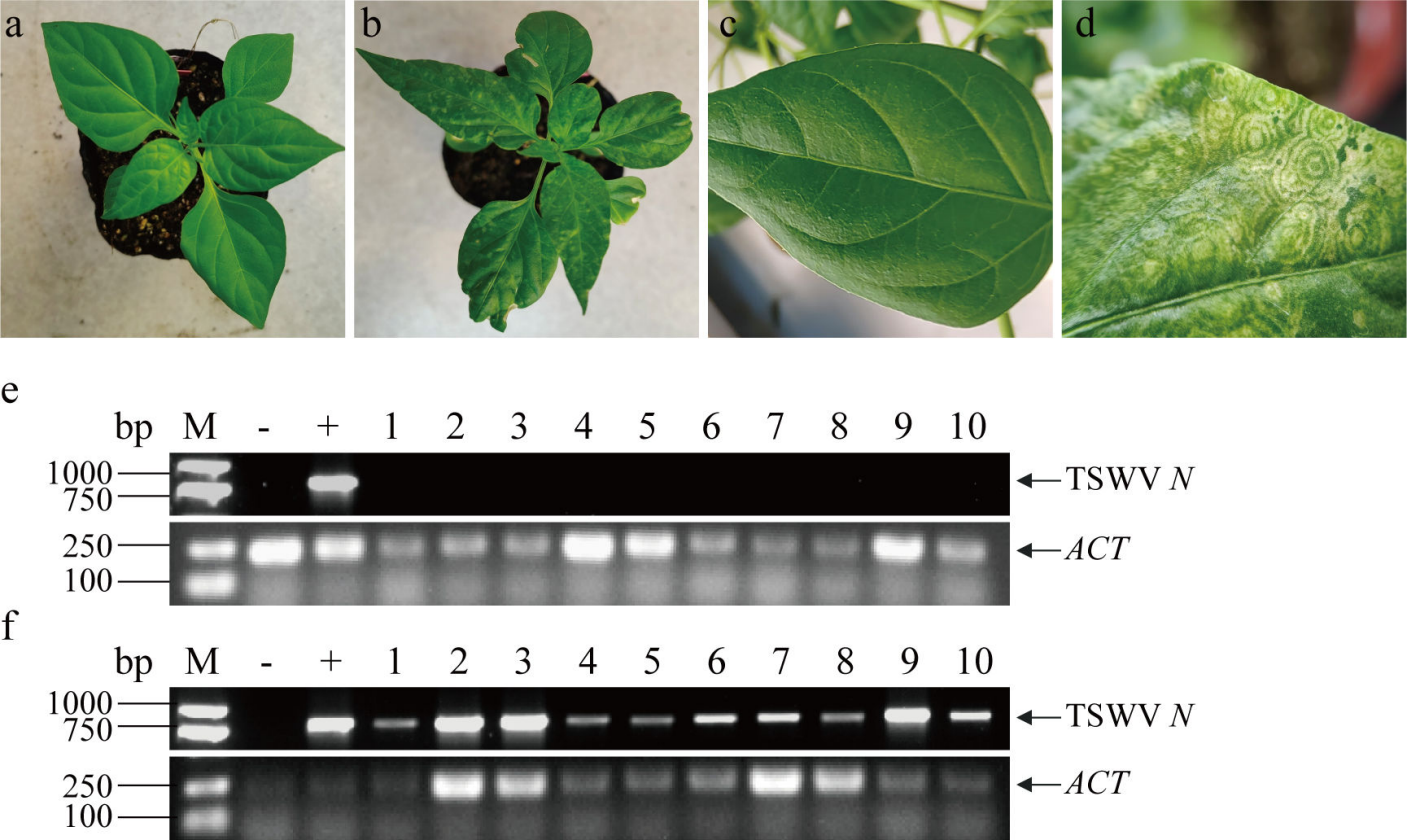
Symptoms and RT-PCR detection of tomato spotted wilt virus (TSWV) in pepper plants at different stages. Pepper plants were inoculated for 2 weeks with either inoculation buffer (**a**) or TSWV (**b**). After 2 months, leaves from buffer-inoculated (**c**) and TSWV-inoculated (**d**) plants were examined for symptom development. RT-PCR detection of TSWV-*N* and pepper *ACT* in systemic leaves. (**e**) Control plants showing no TSWV-*N* amplification; (**f**) TSWV-inoculated plants showing viral amplification in multiple samples. M: DNA ladder (2,000 bp); -, negative control; +, positive control; 1–10, individual plant samples. *ACT* was used as an internal reference gene.

Two weeks post-inoculation, TSWV-inoculated plants exhibited chlorosis and leaf curling in emerging leaves (Fig. 1b), whereas buffer-inoculated control plants remained symptomless (Fig. 1a). Two months later, controls maintained normal morphology (Fig. 1c), while infected plants developed chlorosis and ring spot patterns (Fig. 1d). Systemic infection was confirmed in emerging leaves by RT-PCR detection of the approximately 777-bp TSWV-*N* gene amplicon (Fig. 1f, lanes f1–f10); no product was detected in the equivalent healthy control samples (Fig. 1e, lanes e1–e10). Amplification of the pepper *ACT* (~228 bp) transcript verified cDNA quality in both infected and healthy samples (Fig. 1e and f).

### Spatial distribution of TSWV virions in *C. annuum* reproductive tissues

**Fig 2:**
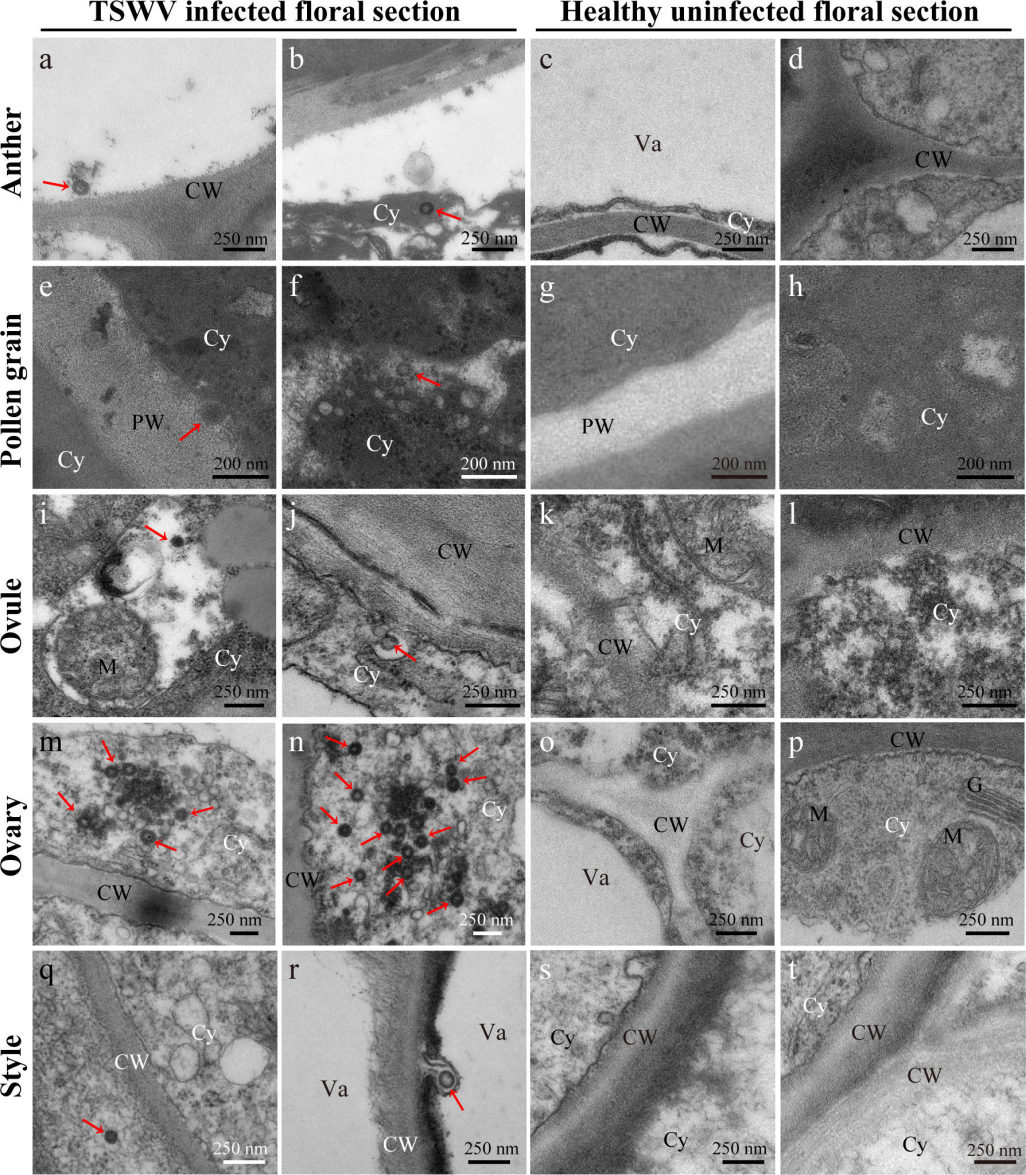
Spatial distribution of tomato spotted wilt virus (TSWV) virions in *C. annuum* reproductive tissues. Transmission electron microscopy images show enveloped spherical TSWV virions (red arrows; 80–120 nm) in infected (**a, b, e, f, i, j, m, n, q, r**) and their absence in healthy (**c, d, g, h, k, l, o, p, s, t**) floral tissues of chili pepper. Va, vacuole; CW, cell wall; PW, pollen wall; M, mitochondria; Cy, cytoplasm. Scale bars are indicated in each image.

**Fig 3:**
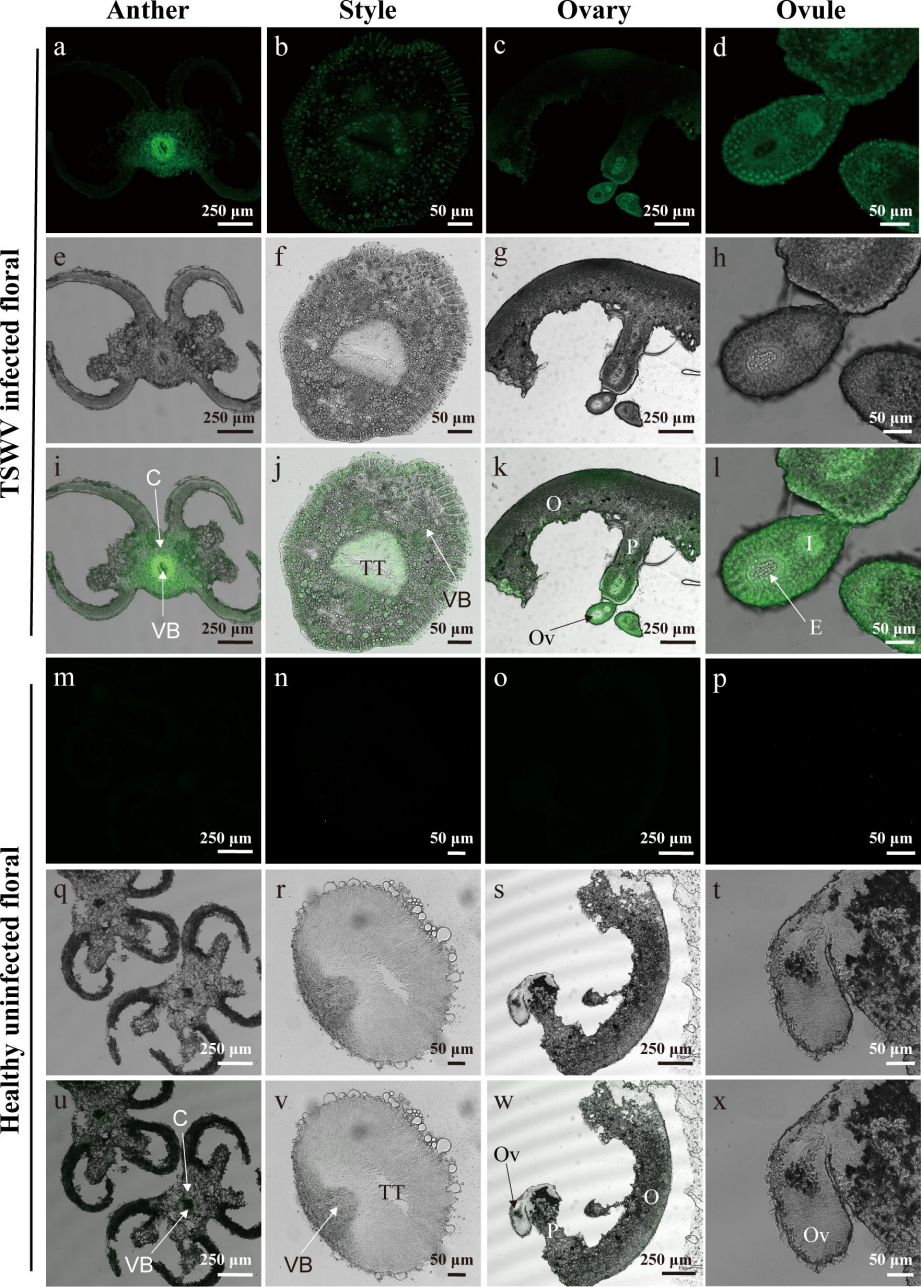
Localization of tomato spotted wilt virus (TSWV) nucleocapsid N protein in reproductive tissues of *C. annuum*. Floral tissues from TSWV-infected and healthy pepper plants were analyzed under bright-field and fluorescence microscopy. Sections were incubated with a mouse monoclonal primary antibody against TSWV N, followed by a fluorescently labeled goat anti-mouse secondary antibody (488 nm channel). Images show anthers (**a, e, i**), styles (**b, f, j**), ovaries (**c, g, k**), and ovules (**d, h, l**) from TSWV-infected plants and corresponding tissues from healthy controls: anthers (**m, q, u**), styles (**n, r, v**), ovaries (**o, s, w**), and ovules (**p, t, x**). Bright-field images are presented in panels **e–h** and **q–t**, fluorescence images in **a–d** and **m–p**, and overlay images in **i–l** and **u–x**. Fluorescence signals in infected tissues indicate the presence of TSWV N protein, particularly in reproductive structures such as ovules (**a–d**). In the control group, no fluorescence was detected (**m–p**). Structural annotations: C, connective; VB, vascular bundle; TT, transmitting tissue; O, ovary wall; P, placenta; Ov, ovule; I, integument; E, embryo sac. Scale bars are indicated in each image.

**Fig 4:**
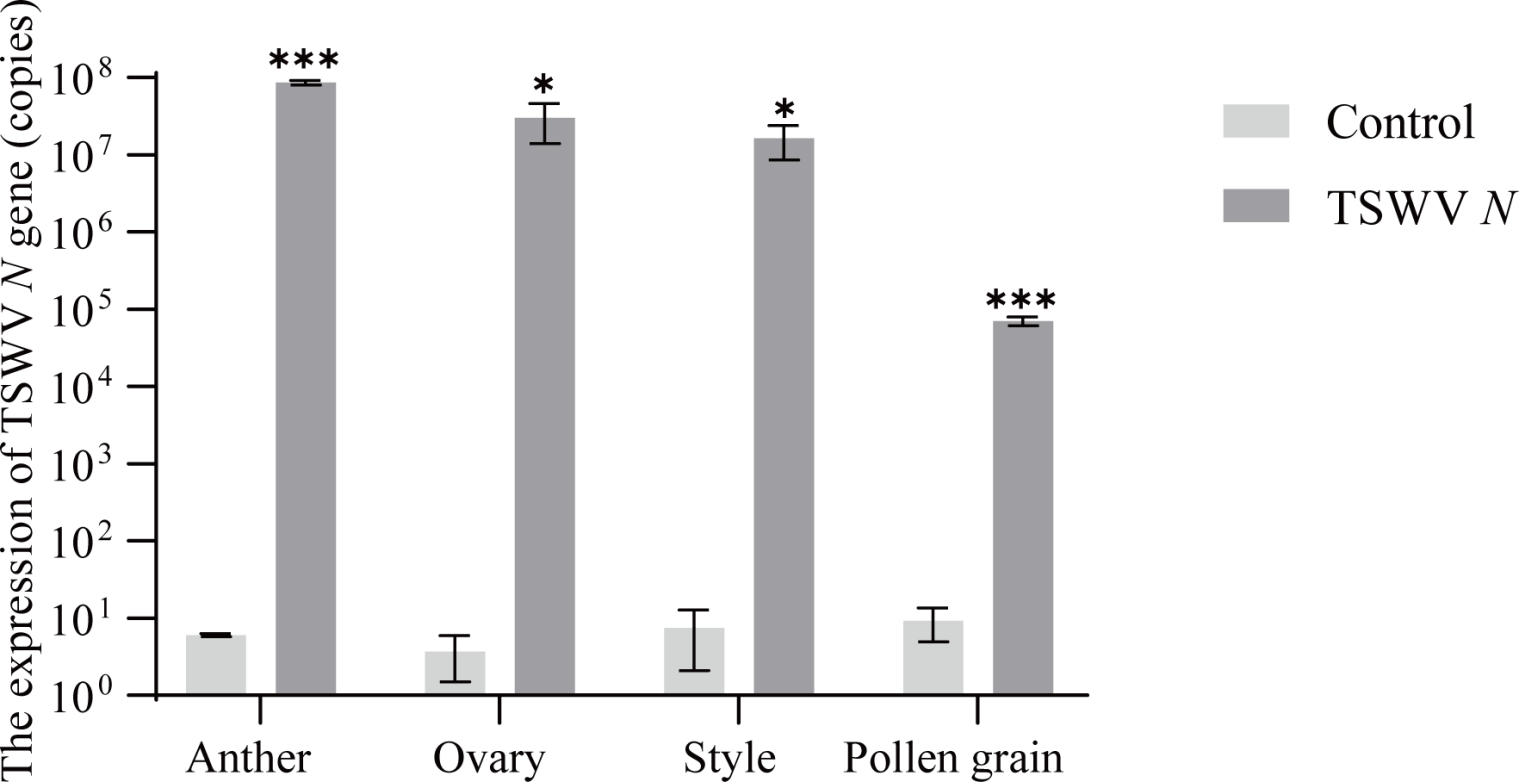
Absolute RNA levels of the TSWV nucleocapsid (*N*) gene in anthers, ovaries, styles, and pollen grains of pepper plants. Values represent mean ± SD (*n* = 3). Statistical significance between infected and mock-inoculated controls was analyzed using a two-tailed Student’s *t*-test (GraphPad Prism v9.0). *P < 0.05, **P < 0.01, ***P < 0.001.

TEM of ultrathin sections from anthers, pollen grains, ovaries, ovules, and styles of infected pepper plants revealed TSWV-like enveloped spherical particles measuring 80–100 nm in diameter, which is consistent with the reported particle diameter range for TSWV (65–135 nm) (Fig. 2). The virions were predominantly localized in the cytoplasmic matrix of infected anthers (Fig. 2a and b) and within the cytoplasm and at the interface between the intine and protoplast in pollen grains (Fig. 2e and f). In ovules, virions accumulated in integument cells (Fig. 2i and j). Ovaries contained high-density virion clusters and viroplasm-like inclusions consistent with active replication and assembly (Fig. 2m and n), while styles displayed both intracellular virions and vesicle-like structures near the cell wall (Fig. 2q and r). However, in the control tissues, no particles with the characteristic size, envelope, and clustering patterns of TSWV were observed across multiple fields of view (Fig. 2c, d, g, h, k, l, o, p, s, and t). Occasional electron-dense vesicles were present in control tissues but differed in morphology and were not accompanied by corroborating N-protein labeling or viral RNA detection (Fig. 3 and 4). Together, these observations support systemic colonization of reproductive organs, consistent with vertical transmission through pollen and ovule-associated routes.

### Localization of TSWV-N protein in reproductive tissues of *C. annuum*

Immunofluorescence histochemistry confirmed accumulation of the TSWV-N protein in reproductive tissues of *C. annuum* flowers. The TSWV-infected flowers exhibited intense TSWV-N-specific immunolabeling in anthers, styles, ovaries, and ovules (Fig. 3a through d), while maximal accumulation occurred in the anther septa and vascular bundles (Fig. 3a and i). Additionally, styles displayed significant labeling in vascular bundles and transmittingtissue (Fig. 3b and j). The ovary wall and placenta exhibited intense fluorescence, indicating pervasive viral infiltration (Fig. 3c and k), while healthy controls showed no detectable signals (Fig. 3m through p). The pronounced accumulation of the TSWV-N proteins within the ovule, particularly the intense fluorescence in the integuments, suggests the ovule’s potential involvement in the seed transmission pathways (Fig. 3d and l).

### TSWV *N* gene expression in the reproductive tissues of *C. annuum*

To investigate the distribution of TSWV in reproductive tissues, TSWV *N* RNA was quantified by RT-qPCR using a plasmid-based absolute standard. TSWV *N* RNA was readily detected in anthers, ovaries, styles, and pollen grains of infected plants, whereas it was undetectable or near the limit of detection in mock-inoculated controls. Based on absolute quantification, TSWV *N* gene copy numbers (copies μL^−1^) in anthers, ovaries, and styles were >10^7^-fold higher than in mock-inoculated controls, whereas those in pollen grains approached 10^5^-fold (Fig. 4).

### Seed transmission of TSWV through pollen- and ovules-associated routes in *C. annuum*

**Fig 5:**
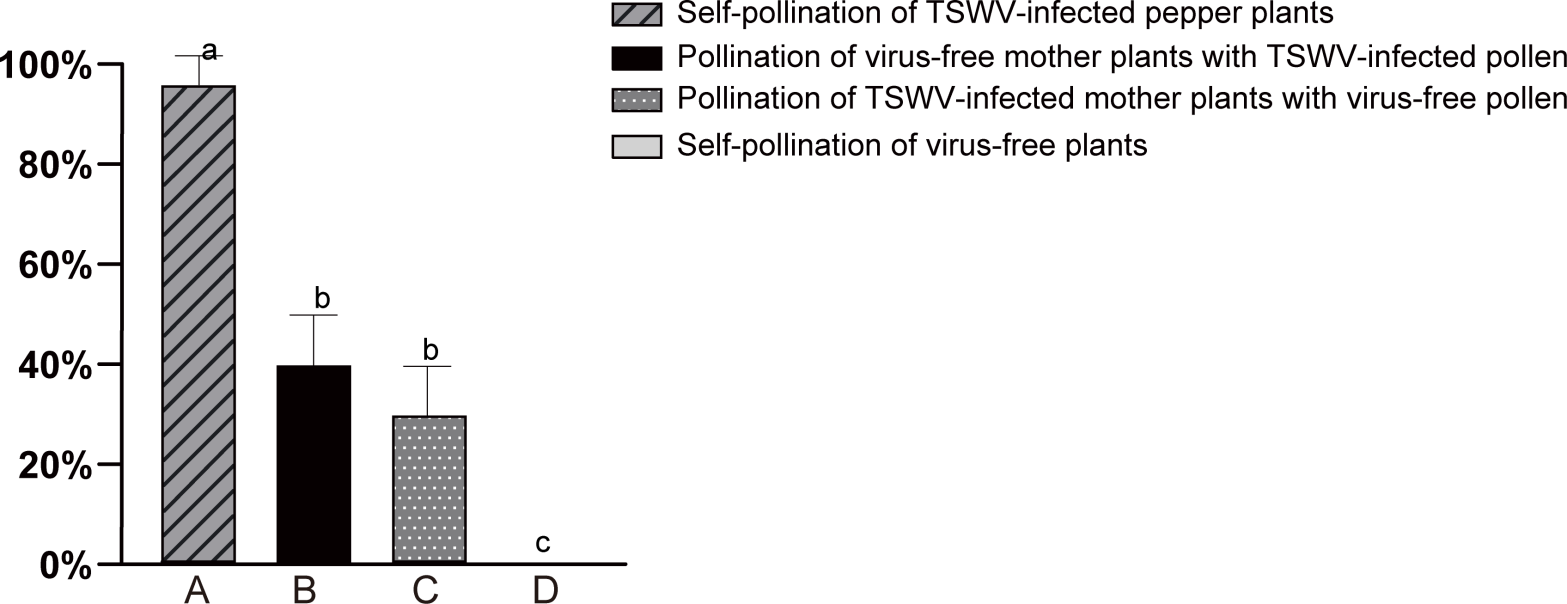
The rate of tomato spotted wilt virus (TSWV) detection in seeds resulting from four different pollination combinations. Individual seeds were surface-disinfected, homogenized, and tested for TSWV by RT-PCR targeting the *N* gene as described in Materials and Methods. Values represent the mean ± SD (*n* = 3). The four cross-pollination treatments were (A) self-pollination of infected plants with infected pollen; (B) pollination of virus-free mother plants with infected pollen (pollen-associated transmission); (C) pollination of TSWV-infected mother plants with virus-free pollen (ovule/maternal-associated transmission); (D) self-pollination of virus-free plants. Different lowercase letters (a, b, c) above the bars indicate statistically significant differences among treatments (*P* < 0.05). Treatments sharing the same letter are not significantly different from each other.

To evaluate vertical transmission via pollen and ovules, we performed cross-pollination treatments: (i) self-pollination of TSWV-infected plants, (ii) pollination of virus-free maternal plants with infected pollen, (iii) pollination of infected maternal plants with virus-free pollen, and (iv) self-pollination of virus-free controls. These treatments resulted in seed infection rates of 96%, 40%, 30%, and 0%, respectively (Fig. 5; Fig. S1). Thus, both infected pollen and infected maternal tissues/ovules can contribute to vertical transmission. We further tested progeny seedlings derived from each treatment and detected TSWV infection rates of 90% in self-pollination of infected plants, 20% pollen-mediated transmission, and 30% ovule-mediated transmission (Fig. S2). Therefore, these data confirm that TSWV is transmitted vertically via gametophytic (pollen) and sporophytic (ovule) pathways, highlighting reproductive organs as critical epidemiological reservoirs in pepper agroecosystems.

## DISCUSSION

**Fig 6:**
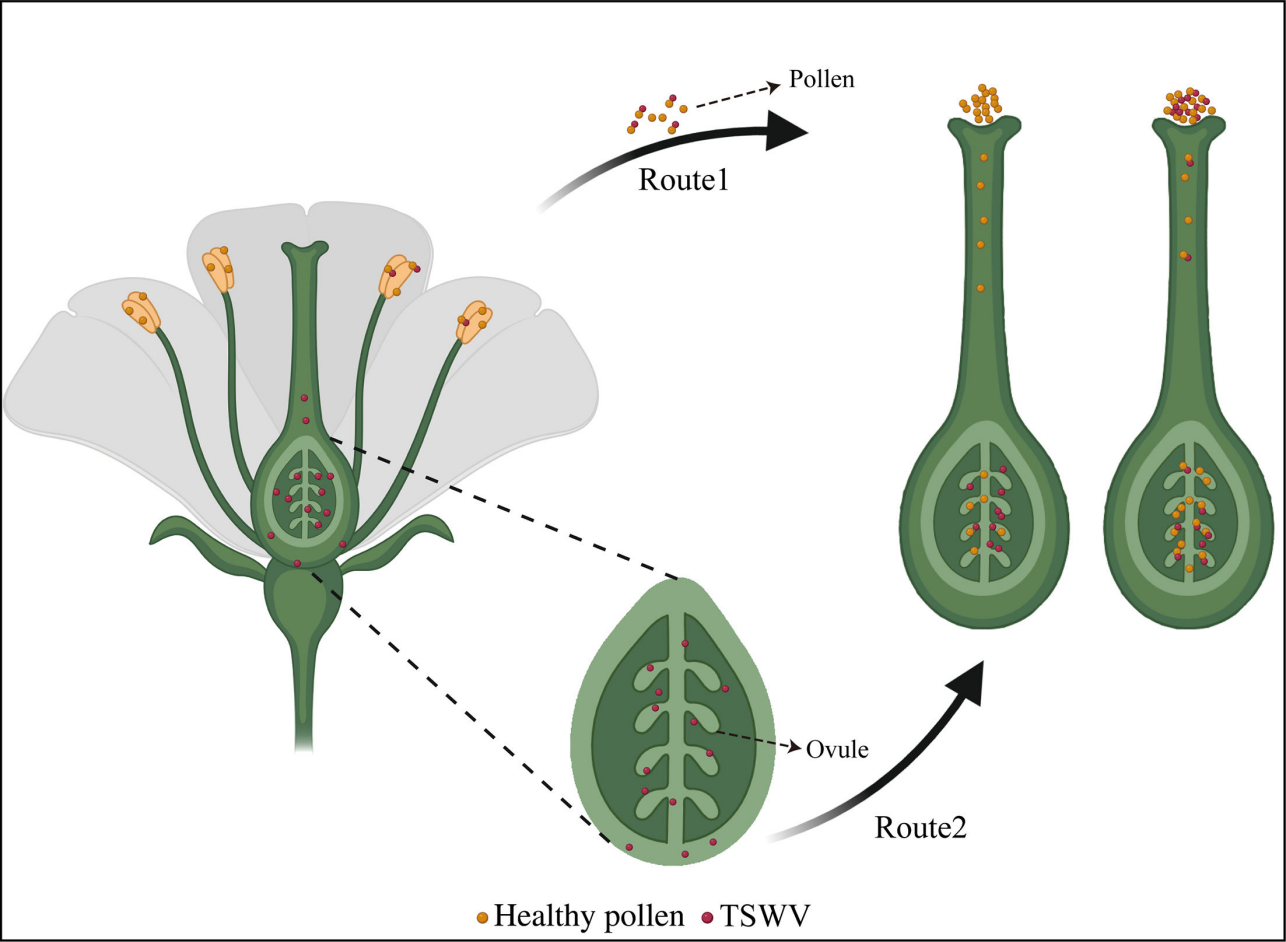
Schematic representation of vertical transmission mechanisms for tomato spotted wilt virus (TSWV) in *C. annuum*. Two contributing routes within vertical transmission are illustrated: route 1, pollen-associated transmission, where TSWV present in pollen grains has access to the female gametophyte during fertilization; and route 2, maternal/ovule-associated transmission, where infected maternal tissues contribute to progeny, helping explain seed-borne persistence of TSWV in pepper populations.

Seed transmission of plant viruses represents an evolutionary adaptation arising from long-term virus-host co-evolution and involves genotype-dependent interactions between viral pathogens and host plants. This process is modulated by environmental variables (28–30). Therefore, in this study, we mapped TSWV distribution in reproductive tissues and identified its systemic colonization of key reproductive structures, including pollen grains and ovules. Controlled pollination assays further demonstrated that vertical transmission in pepper can be achieved through two reproductive routes: via infected pollen and via infected maternal tissues/ovules (Fig. 6). These findings highlight reproductive organs as key reservoirs supporting seed-borne spread in the pepper production system.

TSWV infection severely compromises the morphology and reproductive efficiency of *C. annuum*, with infected flowers displaying distinct phenotypic abnormalities, including petal edge curling and stunted growth. The viral infection also significantly impairs fruit development, leading to malformation and a marked reduction in seed set compared to healthy controls. In this study, artificial pollination assays using TSWV-infected pollen confirmed these aberrations, as the recipient plants produced deformed fruits (20, 31). These observations suggest that TSWV disrupts reproductive organ development, potentially by interfering with hormonal regulation or cellular integrity during gametogenesis and fertilization.

TSWV exhibits tissue-specific tropism within the reproductive organs of *C. annuum*, with higher accumulation in anthers, ovaries, and styles than in mature pollen grains, suggesting differential viral replication efficiency between these tissues, which likely reflects distinct biological constraints. Studies suggest that anthers provide a nutrient-rich microenvironment conducive to viral replication, while mature pollen grains exhibit restricted viral replication because of limited cellular resources and physical barriers imposed by their developing pollen walls (28). Therefore, based on these findings, we hypothesize that TSWV likely enters pollen during early developmental stages, before sclerification, through cell-to-cell movement from infected anther tissues into developing pollen cells.

During seed development in *C. annuum*, the ovule undergoes coordinated differentiation, in which integuments form the seed coat, fertilized polar nuclei develop into endosperm, and the zygote gives rise to the embryo. In this study, immunofluorescence histochemical results demonstrated intense fluorescent signals corresponding to TSWV in the integuments, suggesting substantial viral accumulation in the seed coat tissues of *C. annuum*. However, this observation requires further experimental validation through quantitative assays. Previous research on TSWV seed transmission has primarily focused on viral accumulation in endosperm tissues, suggesting that the endosperm serves as a primary reservoir for vertical transmission (20). Interestingly, seed coat-associated viral transmission has been reported for the tobamovirus tomato brown rugose fruit virus (ToBRFV) (29). Whether a similar mechanism contributes to TSWV seed transmission remains to be determined.

Simultaneously quantifying paternal and maternal contributions to seed-borne TSWV, we found that infected pollen resulted in 40% seed infection but only 20% of first filial (F1) generation seedlings were infected, whereas infected ovules led to 30% seed infection with consistent transmission to F1 seedlings (30%). Systemically infected plants undergoing self-pollination (both pollen and ovules infected) exhibited 96% seed infection (90% in F1 seedlings). This finding demonstrates that either gamete can independently mediate vertical transmission, with pollen delivering a higher initial viral load, while the maternal pathway provides a more persistent, stably inherited inoculum that is consistently recovered in the next generation. All F1 progeny remained symptomless despite systemic infection (Fig. S3), indicating that the virus can be seed transmitted and subsequently masked by plant immunity. Such cryptic carriers could evade visual inspection and even late-stage molecular testing, underscoring the need to include pollen-targeted qPCR in seed-certification protocols to prevent covert TSWV dissemination.

To date, seed transmission of orthotospoviruses remains controversial. Early reports described TSWV seed transmission in tomato (22, 23), but subsequent studies questioned this (24) or failed to detect the virus in tomato seeds (25). In pepper, previous work detected TSWV in the endosperm but not the embryo, with only transient positivity in seedlings (20). Here, we provide direct evidence that TSWV is seed transmitted in *C. annuum* via both pollen and ovules and that the virus can establish systemic infection in the next generation, albeit asymptomatically. In subsequent studies, it remains to be elucidated whether seed-transmitted TSWV persists across multiple generations and whether similar vertical transmission occurs in tomato. Considering that tomato and pepper both belong to the *Solanaceae* family but exhibit divergent reproductive traits and virus susceptibility, further research is warranted to determine whether TSWV colonizes tomato reproductive tissues and achieves vertical transmission.

In conclusion, our study provides evidence that TSWV seed transmission in pepper can occur through both pollen-associated and maternal/ovule-associated routes, establishing a foundation for developing targeted strategies to reduce seed-borne viral spread. Further research should dissect the molecular mechanisms governing viral movement into reproductive tissues and determine how infection timing and tissue barriers shape transmission efficiency. Such mechanistic insights will support genetic and management interventions to mitigate seed transmission in *C. annuum* and other economically important crops. By integrating fundamental virology with applied crop protection strategies, this work also paves the way for sustainable management of TSWV in agricultural systems.

## MATERIALS AND METHODS

### Plant materials

The chili pepper cultivar *Changyan*, susceptible to TSWV, was cultivated in a controlled phytotron environment at 25°C with 30% relative humidity and a 16-h light/8-h dark photoperiod. Plants were grown in batches, with 20 plants sown every 2 weeks to ensure a continuous supply of experimental material.

### TSWV inoculation

The TSWV isolate 20YV181 (GenBank: OP893534), originally obtained from infected pepper plants in Changdu, Xizang, China (31.1°−31.2°N, 97.1°−97.3°E), was maintained on *Nicotiana benthamiana* through mechanical inoculation. *C. annuum* had 4–5 true leaves that were slightly dusted with quartz sand and then mechanically inoculated with TSWV as described (17, 30). The control plants were mock inoculated with inoculation buffer: 33 mM KH_2_PO_4_, 87.8 mM K_2_HPO_4_, and 10 mM Na_2_SO_3_. Each experimental group consisted of 10 pepper plants.

### Reproductive organ isolation

Floral reproductive structures, including styles, ovaries, anthers, and pollen grains, were carefully dissected from approximately 6–7 flowers per plant from 10 TSWV-infected and 10 uninfected pepper plants using a dissection needle under a stereomicroscope. After dissection, the tissues from each type of floral reproductive structure were pooled together to create mixed samples for subsequent experiments.

### RNA extraction and RT-PCR detection of TSWV in leaves

Total RNA was isolated from 50 mg of pepper leaf tissue using the MiniBEST Plant RNA Extraction Kit (TaKaRa, Kusatsu-Shiga, Japan) and reverse transcribed with 5× EasyQuick RT MasterMix (CWBIO, Jiangsu, China). For routine detection, a 777-bp fragment of the TSWV nucleocapsid (*N*) gene was amplified by RT-PCR with primers TSWV *N*-F (5′-ATGTCTAAGGTTAAGCTCACTA-3′) and TSWV *N*-R (5′-TTAAGCAAGTTCTGCAAGTT-3′). To monitor RNA/cDNA quality, the pepper housekeeping gene *ACT* (228 bp) was amplified in parallel as an internal control with primers *ACT*-Actin-F (5′-TGTTATGGTAGGGATGGGTC-3′) and *ACT*-Actin-R (5′-TTCTCTCTATTTGCCTTGGG-3′).

### RNA extraction and RT-qPCR for floral organs

Ovaries, styles, and anthers were processed as above after collecting an equal mass of each tissue; pollen was pre-digested with cellulase to disrupt the thick wall before RNA extraction (32). Quantitative PCR targeted a 166-bp fragment of the TSWV-*N* gene using primers 5′-GGAGCCACTGACATGACCTT-3′ and 5′-GCCTCACAGACTTTGCATCA-3′ with Hieff qPCR SYBR Green Master Mix on an ABI QuantStudio 6 system (95°C for 2 min; 40 cycles of 95°C for 10 s, 60°C for 20 s). A 10-fold dilution series of the TSWV*-N* plasmid (76.1 ng μL^−1^) generated the standard curve for absolute quantification.

### Transmission electron microscopy

After pre-embedding pollen grains in agarose, small tissue segments measuring 1 × 1 mm of pollen grains, anthers, styles, and ovaries were collected from both TSWV-infected and uninfected plants and fixed in 2.5% glutaraldehyde at 4°C for 12 h as described previously (33). The samples were dehydrated using standard protocols (34). Ultrathin sections of 70 nm were prepared using a Leica EM UC7 ultramicrotome (Leica, Wetzlar, Germany), stained with 2% uranyl acetate and lead citrate, and examined under an FEI TECNAI Spirit G2 TEM at 80 kV. For each tissue type, 10–15 fields of view were examined.

### Immunohistochemistry (IHC)

The ovaries, anthers, and styles (approximately 2 × 2 × 1 mm^3^) were fixed in PBS containing 4% paraformaldehyde and 0.1% glutaraldehyde, subjected to sucrose gradient infiltration (35), and immunofluorescently labeled (35, 36). Samples were incubated with a mouse monoclonal antibody against TSWV N protein (provided by Prof. X. Tao, Nanjing Agricultural University, Nanjing, China), followed by incubation with an Alexa Fluor 488–conjugated goat anti-mouse IgG secondary antibody (Abcam, Cambridge, UK). Labeled samples were observed under a laser confocal laser microscope (Leica TCS-SP8). Each sample was prepared with at least three slides containing 3 to 5 sections for field observation.

### Cross-pollination experiments

**Fig 7:**
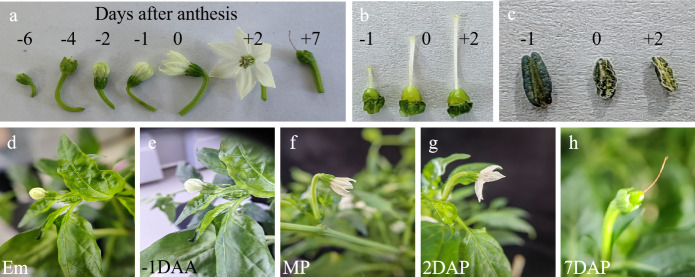
The phenotype of style and ovary during the flowering stage in pepper and the illustration of the timing for manual pollination. (**a**) Flowering process; (**b**) ovary and style (style length: 3, 7, 10 mm); (**c**) anther (4, 3, 2 mm); (**d**) 2 days before anthesis; (**e**) 1 day before anthesis; (**f**) anthesis; (**g**) 2 days after anthesis (day 2 of pollination); (**h**) 7 days after anthesis (day 7 of pollination), showing ovary expansion and flower wilting. Em, emasculation; DAA, days after anthesis; MP, manual pollination; DAP, days after pollination. In panels a to c, numbers represent the days after anthesis.

Two days before anthesis (Fig. 7d), anthers were manually removed from both TSWV-infected and uninfected plants. On the flowering day (Fig. 7f), stigmas from healthy plants were pollinated with pollen from TSWV-infected plants, and vice versa. After 7 days of pollination (DAP), styles wilted, while the ovaries began to swell (Fig. 7).

### Detection of TSWV infection in seeds and seedlings

After fruit maturity at 45 days after pollination (45 DAP), 30 seeds were collected from each self and cross-pollinated treatment. The seeds were soaked in water for 3–4 h, disinfected with 10% (wt/vol) trisodium phosphate for 20–30 min, and rinsed thoroughly with sterile water until their pH reached 7.0 (37–39). Individual seeds were flash-frozen in liquid nitrogen and homogenized using a cryogenic tissue grinder (JXESTPRP-CLN-481, Shanghai Jingxin Industrial Development Co., Ltd., Shanghai, China) at −20°C for 90 s at 30 Hz. The seedling detection method is described in “RNA extraction and RT-qPCR,” above. Total RNA was isolated from 30-day-old pepper seedlings (two-true-leaf stage, ca. 1 month postgermination) and assayed by RT-PCR using the TSWV-specific primer set TSWV *N*-F/R. To monitor RNA/cDNA quality, the pepper housekeeping transcript ACT was amplified in parallel as an internal control with primers ACT-ActinF/R.

### Statistical analysis

Absolute transcript copy numbers were calculated according to the method of Li et al. (40). RT-qPCR data are presented as mean ± SD from three biological replicates. For cross-pollination assays, differences in TSWV detection rates among pollination treatments were assessed by one-way analysis of variance followed by Duncan’s multiple range test. Different lowercase letters above bars indicate statistically significant differences (*P* < 0.05). Data are presented as means ± standard deviations (*n* = 3).

## Data Availability

The genomic sequence of the Tomato spotted wilt virus (TSWV) isolate 20YV181 used in this study has been deposited in the GenBank database under accession number OP893534. All other relevant data generated or analyzed during this study are included in the article and its supplemental material. Software and databases used during this study include NCBI BLAST (https://blast.ncbi.nlm.nih.gov/), GenBank (https://www.ncbi.nlm.nih.gov/genbank/), and GraphPad Prism (https://www.graphpad.com/scientific-software/prism/). Additional data are available from the corresponding author upon reasonable request.

## SUPPLEMENTAL MATERIAL

Supplemental material jvi.00223-26-s0001.doc Fig. S1 to S3.

ASM does not own the copyrights to Supplemental Material that may be linked to, or accessed through, an article. The authors have granted ASM a non-exclusive, world-wide license to publish the Supplemental Material files. Please contact the corresponding author directly for reuse.

## References

[1] Sastry KS. 2013. Seed transmission of viruses. In Sastry KS (ed), Seed-borne plant virus diseases. Springer, Delhi.

[2] Jones RAC, Congdon BS. 2024. Australian cool-season pulse seed-borne virus research: 1. Alfalfa and cucumber mosaic viruses and less important viruses. Viruses 16:144. doi:10.3390/v16010144 38257844 PMC10819373

[3] Kumari N, Sharma V, Patel P, Sharma PN. 2023. Pepper mild mottle virus: a formidable foe of capsicum production—a review. Front Virol 3:1208853. doi:10.3389/fviro.2023.1208853

[4] Escalante C, Sanz-Saez A, Jacobson A, Otulak-Kozieł K, Kozieł E, Balkcom KS, Zhao C, Conner K. 2024. Plant virus transmission during seed development and implications to plant defense system. Front Plant Sci 15:1385456. doi:10.3389/fpls.2024.1385456 38779063 PMC11109449

[5] Sandra N, Mandal B. 2024. Emerging evidence of seed transmission of Begomoviruses: implications in global circulation and disease outbreak. Front Plant Sci 15:1376284. doi:10.3389/fpls.2024.1376284 38807782 PMC11130427

[6] Zhang ZK, Zheng KY, Zhao LH, Su XX, Zheng X, Wang TT. 2021. Occurrence, distribution, evolutionary relationships, epidemiology, and management of Orthotospoviruses in China. Front Microbiol 12:686025. doi:10.3389/fmicb.2021.686025 34421843 PMC8371445

[7] Tiberini A, Cillo F, Gentili A, Bertin S. 2025. Tomato spotted wilt virus (Orthotospovirus tomatomaculae), a cyclically occurring threat to crop production worldwide. Ann Appl Biol 186:93–114. doi:10.1111/aab.12977

[8] Pappu HR, Jones RA, Jain RK. 2009. Global status of tospovirus epidemics in diverse cropping systems: successes achieved and challenges ahead. Virus Res 141:219–236. doi:10.1016/j.virusres.2009.01.009 19189852

[9] Scholthof KB, Adkins S, Czosnek H, Palukaitis P, Jacquot E, Hohn T, Hohn B, Saunders K, Candresse T, Ahlquist P, Hemenway C, Foster GD. 2011. Top 10 plant viruses in molecular plant pathology. Mol Plant Pathol 12:938–954. doi:10.1111/j.1364-3703.2011.00752.x 22017770 PMC6640423

[10] Zhu M, van Grinsven IL, Kormelink R, Tao XR. 2019. Paving the way to tospovirus infection: multilined interplays with plant innate immunity. Annu Rev Phytopathol 57:41–62. doi:10.1146/annurev-phyto-082718-100309 30893008

[11] Kormelink R, Garcia ML, Goodin M, Sasaya T, Haenni AL. 2011. Negative-strand RNA viruses: the plant-infecting counterparts. Virus Res 162:184–202. doi:10.1016/j.virusres.2011.09.028 21963660

[12] Yvon M, German TL, Ullman DE, Dasgupta R, Parker MH, Ben-Mahmoud S, Verdin E, Gognalons P, Ancelin A, Laï Kee Him J, Girard J, Vernerey MS, Fernandez E, Filloux D, Roumagnac P, Bron P, Michalakis Y, Blanc S. 2023. The genome of a Bunyavirus cannot be defined at the level of the viral particle but only at the scale of the viral population. Proc Natl Acad Sci USA 120:e2309412120. doi:10.1073/pnas.2309412120 37983500 PMC10691328

[13] Oliver JE, Whitfield AE. 2016. The genus Tospovirus: emerging bunyaviruses that threaten food security. Annu Rev Virol 3:101–124. doi:10.1146/annurev-virology-100114-055036 27578436

[14] Li J, Cao L, Zhao YQ, Shen JH, Wang L, Feng MF, Zhu M, Ye YH, Kormelink R, Tao XR, Wang XX. 2025. Structural basis for the activation of plant bunyavirus replication machinery and its dual-targeted inhibition by ribavirin. Nat Plants 11:518–530. doi:10.1038/s41477-025-01940-y 40044941 PMC11928317

[15] Feng MF, Cheng RX, Chen ML, Guo R, Li LY, Feng ZK, Wu JY, Xie L, Hong J, Zhang ZK, Kormelink R, Tao XR. 2020. Rescue of tomato spotted wilt virus entirely from complementary DNA clones. Proc Natl Acad Sci USA 117:1181–1190. doi:10.1073/pnas.1910787117 31879355 PMC6969498

[16] Zhu M, Jiang L, Bai BH, Zhao WY, Chen XJ, Li J, Liu Y, Chen ZQ, Wang BT, Wang CL, Wu Q, Shen QH, Dinesh-Kumar SP, Tao XR. 2017. The intracellular immune receptor Sw-5b confers broad-spectrum resistance to tospoviruses through recognition of a conserved 21-amino acid viral effector epitope. Plant Cell 29:2214–2232. doi:10.1105/tpc.17.00180 28814646 PMC5635987

[17] Chen J, Zhao YX, Luo XJ, Hong H, Yang TQ, Huang S, Wang CL, Chen HY, Qian X, Feng MF, Chen ZQ, Dong YX, Ma ZC, Li J, Zhu M, He SY, Dinesh-Kumar SP, Tao XR. 2023. NLR surveillance of pathogen interference with hormone receptors induces immunity. Nature 613:145–152. doi:10.1038/s41586-022-05529-9 36517600

[18] Rotenberg D, Jacobson AL, Schneweis DJ, Whitfield AE. 2015. Thrips transmission of tospoviruses. Curr Opin Virol 15:80–89. doi:10.1016/j.coviro.2015.08.003 26340723

[19] Whitfield AE, Ullman DE, German TL. 2005. Tospovirus-thrips interactions. Annu Rev Phytopathol 43:459–489. doi:10.1146/annurev.phyto.43.040204.140017 16078892

[20] Wang HW, Wu XJ, Huang XD, Wei SJ, Lu ZJ, Ye J. 2022. Seed transmission of tomato spotted wilt orthotospovirus in peppers. Viruses 14:1873. doi:10.3390/v14091873 36146680 PMC9504465

[21] Groves C, German T, Dasgupta R, Mueller D, Smith DL. 2016. Seed transmission of Soybean vein necrosis virus: the first Tospovirus implicated in seed transmission. PLoS One 11:e0147342. doi:10.1371/journal.pone.0147342 26784931 PMC4718560

[22] Crowley NC. 1957. Studies on the seed transmission of plant virus diseases. Aust J Biol Sci 10:449–464. doi:10.1071/BI9570449

[23] Jones LK. 1944. Streak and mosaic of cineraria. Phytopathology 34:941–953.

[24] Antignus Y, Lapidot M, Ganaim N, Cohen J, Lachman O, Pearlsman M, Raccah B, Gera A. 1997. Biological and molecular characterization of tomato spotted wilt virus in Israel. Phytoparasitica 25:319–330. doi:10.1007/BF02981095

[25] Atik AK, Paylan İC. 2023. Updating viral agents in tomato seeds with new generation diagnostic technologies. Front Sustain Food Syst 6:945703. doi:10.3389/fsufs.2022.945703

[26] Shanmugam K, Perumal R, Oliva R, Cheng H, Iruthayasamy J, Angappan S, Chandrasekaran IR, Nallusamy S. 2024. Seed transmission of potyviruses: a threat to crop health. Not Bot Horti Agrobo 52:13698. doi:10.15835/nbha52413698

[27] Li L, Wang XF, Zhou GH. 2007. Analyses of maize embryo invasion by Sugarcane mosaic virus. Plant Sci 172:131–138. doi:10.1016/j.plantsci.2006.08.006

[28] Gómez JF, Talle B, Wilson ZA. 2015. Anther and pollen development: a conserved developmental pathway. J Integr Plant Biol 57:876–891. doi:10.1111/jipb.12425 26310290 PMC4794635

[29] Salem NM, Sulaiman A, Samarah N, Turina M, Vallino M. 2022. Localization and mechanical transmission of tomato brown rugose fruit virus in tomato seeds. Plant Dis 106:275–281. doi:10.1094/PDIS-11-20-2413-RE 34293918

[30] Wu Q, Tong C, Chen ZQ, Huang S, Zhao XH, Hong H, Li J, Feng MF, Wang HY, Xu M, Yan YL, Cui HM, Shen DY, Ai G, Xu Y, Li JM, Zhang H, Huang CJ, Zhang ZK, Dong SM, Wang X, Zhu M, Dinesh-Kumar SP, Tao XR. 2023. NLRs derepress MED10b- and MED7-mediated repression of jasmonate-dependent transcription to activate immunity. Proc Natl Acad Sci USA 120:e2302226120. doi:10.1073/pnas.2302226120 37399403 PMC10334756

[31] Roselló S, Díez MJ, Nuez F. 1996. Viral diseases causing the greatest economic losses to the tomato crop. I. The tomato spotted wilt virus — a review. Sci Hortic 67:117–150. doi:10.1016/S0304-4238(96)00946-6

[32] Zhou C, Yang HY. 1989. Experimental manipulation of pollen protoplasts, sperms and generative cells. J Integr Plant Biol 31:726–734.

[33] Zhang ZK, Zheng KY, Dong JH, Fang Q, Hong J, Wang XF. 2016. Clustering and cellular distribution characteristics of virus particles of Tomato spotted wilt virus and Tomato zonate spot virus in different plant hosts. Virol J 13:11. doi:10.1186/s12985-016-0466-x 26786326 PMC4717642

[34] Wang TT, Wei ZL, Xiong ZQ, Wu K, Zhou F, Tong JJ, Jia QQ, Zhu RM, Zhang ZK. 2023. Asparagus lettuce infected with TSWV-the characteristic of virions distribution and subcellular pathological changes. J Chin Electron Microsc Soc 42:53–61. doi:10.3969/j.issn.1000-6281.2023.01.008

[35] Knapp E, Flores R, Scheiblin D, Scheiblin D, Modla S, Czymmek K, Yusibov V. 2012. A cryohistological protocol for preparation of large plant tissue sections for screening intracellular fluorescent protein expression. BioTechniques 52:31–37. doi:10.2144/000113778 22229725

[36] Wan J, Cabanillas DG, Zheng H, Laliberté JF. 2015. Turnip mosaic virus moves systemically through both phloem and xylem as membrane-associated complexes. Plant Physiol 167:1374–1388. doi:10.1104/pp.15.00097 25717035 PMC4378181

[37] Rast ATB, Stijger CCMM. 1987. Disinfection of pepper seed infected with different strains of capsicum mosaic virus by trisodium phosphate and dry heat treatment. Plant Pathol 36:583–588. doi:10.1111/j.1365-3059.1987.tb02277.x

[38] Lapidot M, Guenoune-Gelbart D, Leibman D, Holdengreber V, Davidovitz M, Machbash Z, Klieman-Shoval S, Cohen S, Gal-On A. 2010. Pelargonium zonate spot virus is transmitted vertically via seed and pollen in tomato. Phytopathol 100:798–804. doi:10.1094/PHYTO-100-8-0798 20626283

[39] Harth JE, Simmons HE, Stephenson AG. 2017. Vertical infection of Zucchini yellow mosaic virus via pollen transmission occurs at a lower frequency than ovule transmission. Eur J Plant Pathol 147:717–720. doi:10.1007/s10658-016-1024-5

[40] Li Y, Wu K, Du X, Chen YD, Zhang J, Zhang ZK. 2025. Establishment and application of a SYBR green-based absolute real-time quantitative PCR assay for chilli yellow ringspot virus. J Virol Methods 338:115227. doi:10.1016/j.jviromet.2025.115227 40712892

