# Supplementary material for: Orthotospovirus tomatomaculae (Tomato spotted wilt virus [TSWV]) infects ovules and pollen to achieve vertical transmission in Capsicum annuum
Source: J Virol. 2026 Jun 12;100(7):e00223-26. doi: 10.1128/jvi.00223-26 (PMC13387008; doi:10.1128/jvi.00223-26)
Supplement: Supplemental material — Fig. S1 to S3. [file jvi.00223-26-s0001.doc]

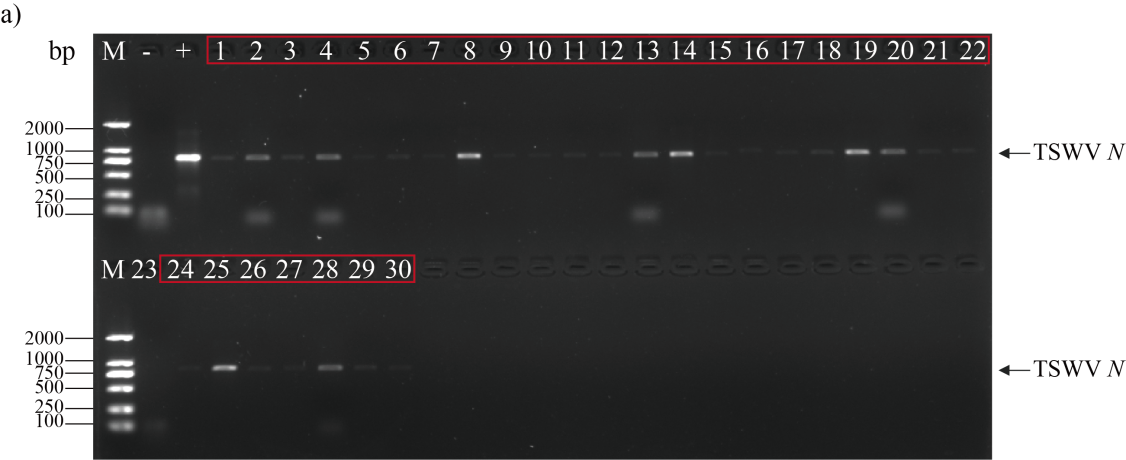

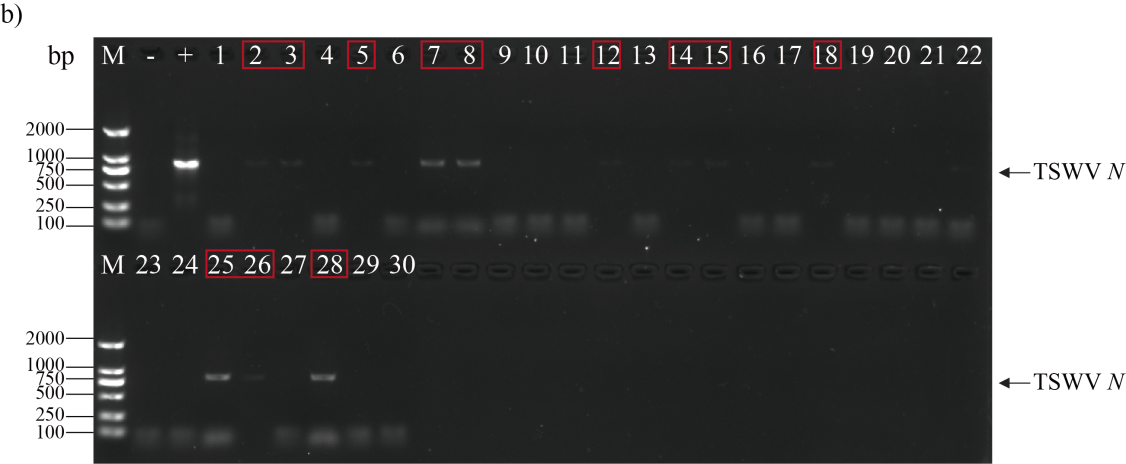

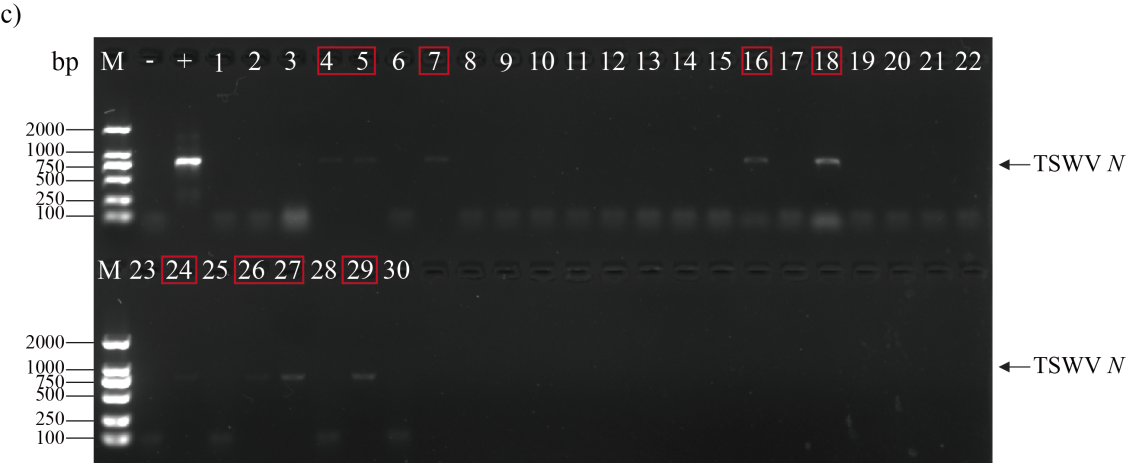

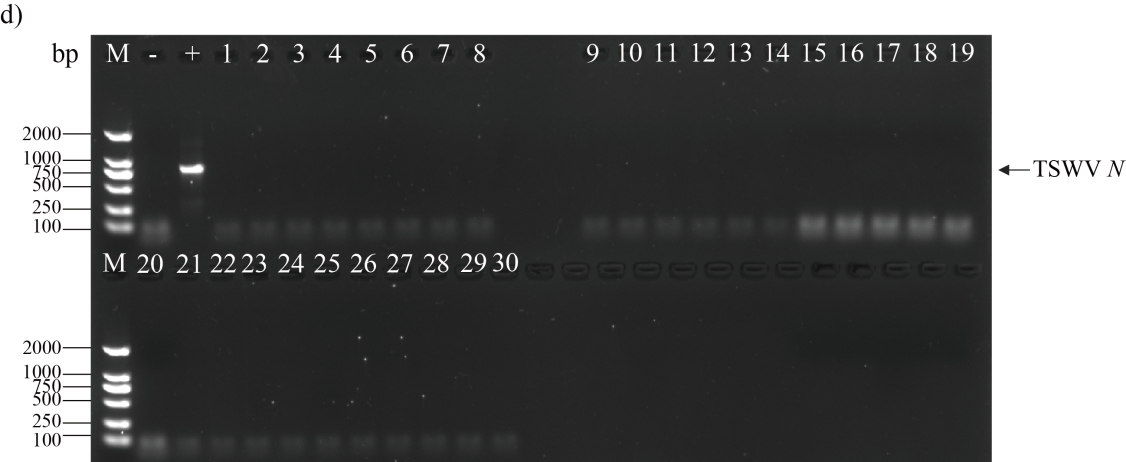

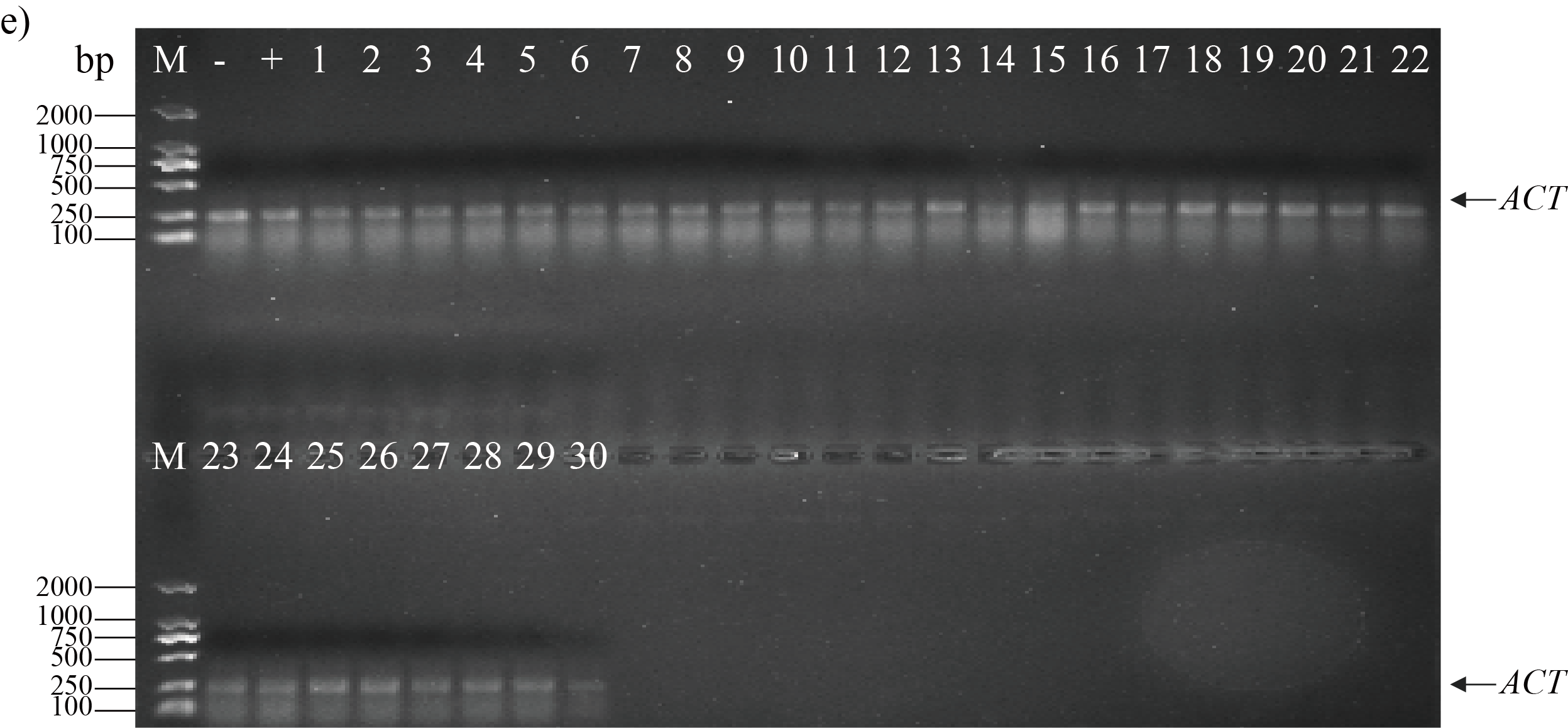

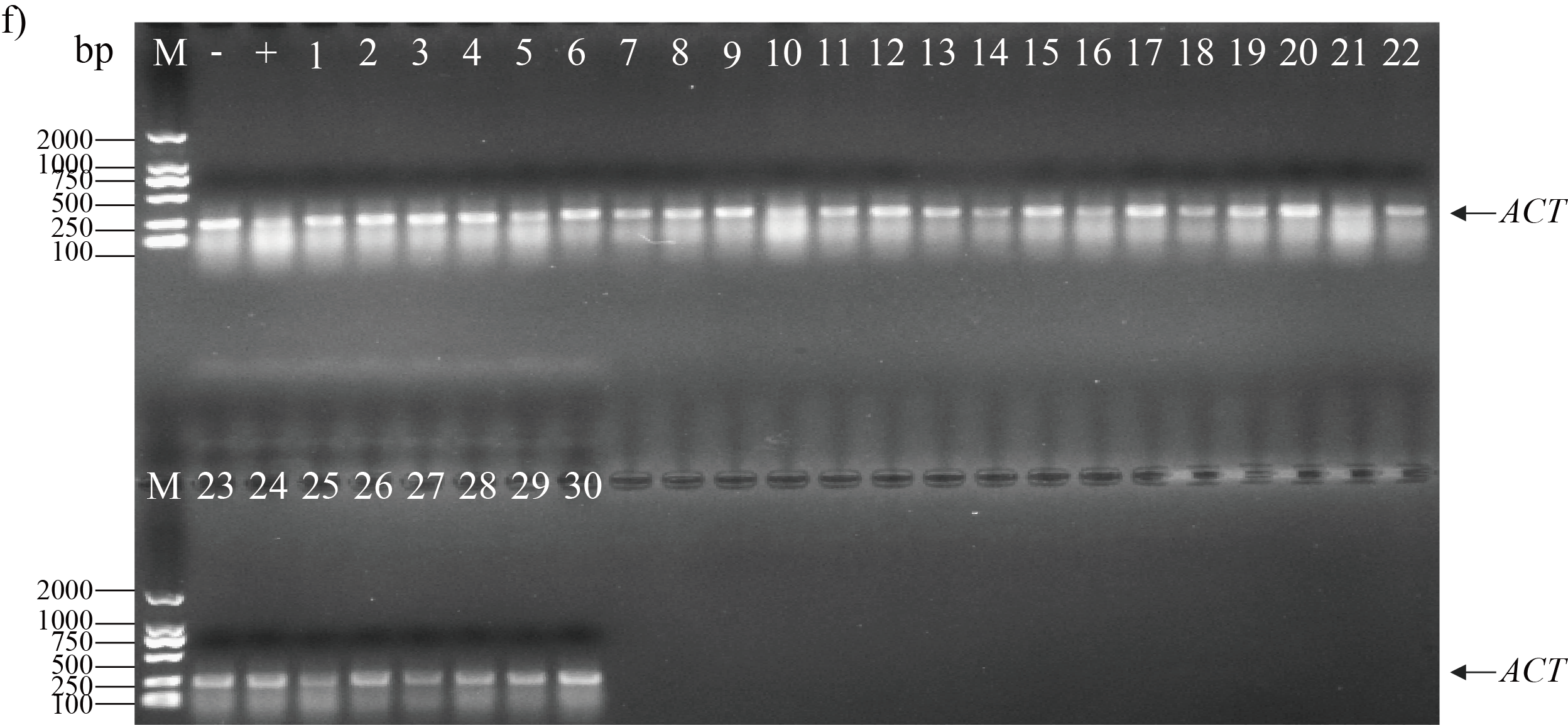

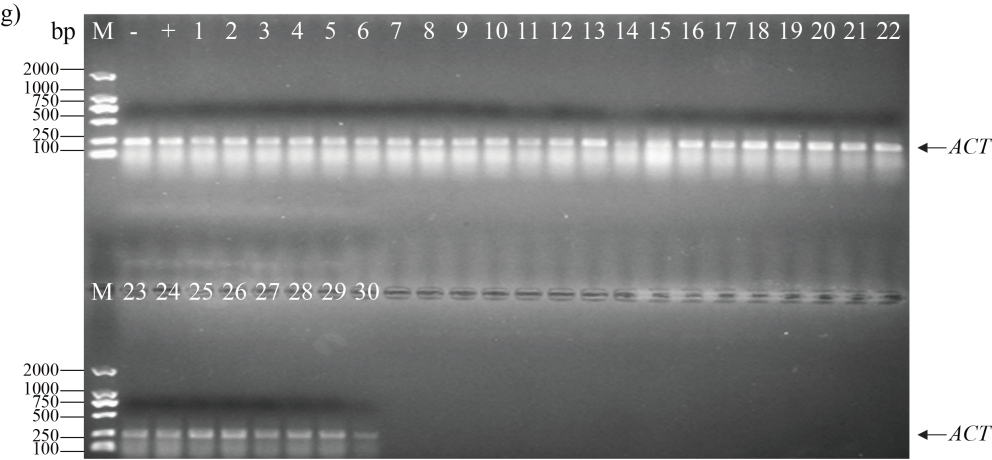

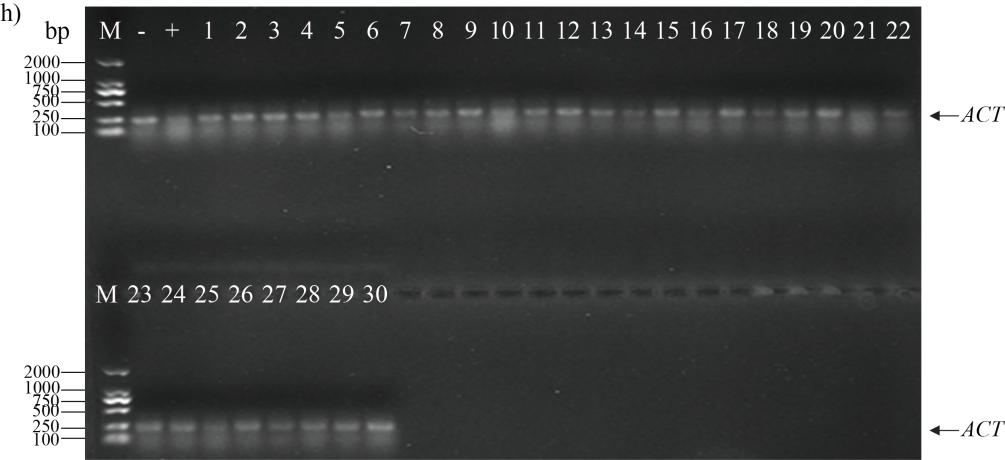


**Supplemental Fig 1** TSWV infection rate of pepper seed under different treatments. RT-PCR was performed to detect the TSWV *N* (777 bp) gene and *ACT* (228 bp) gene in seeds subjected to self-pollination of TSWV-infected pepper plants (a, e), pollination of virus-free mother plants with TSWV-infected pollen (b, f), pollination of TSWV-infected mother plants with virus-free pollen (c, g), and self-pollination of virus-free plants (d, h). M: DNA ladder (2000bp); +, positive control; −, negative control. Red boxes indicate samples in which the TSWV virus was detected.


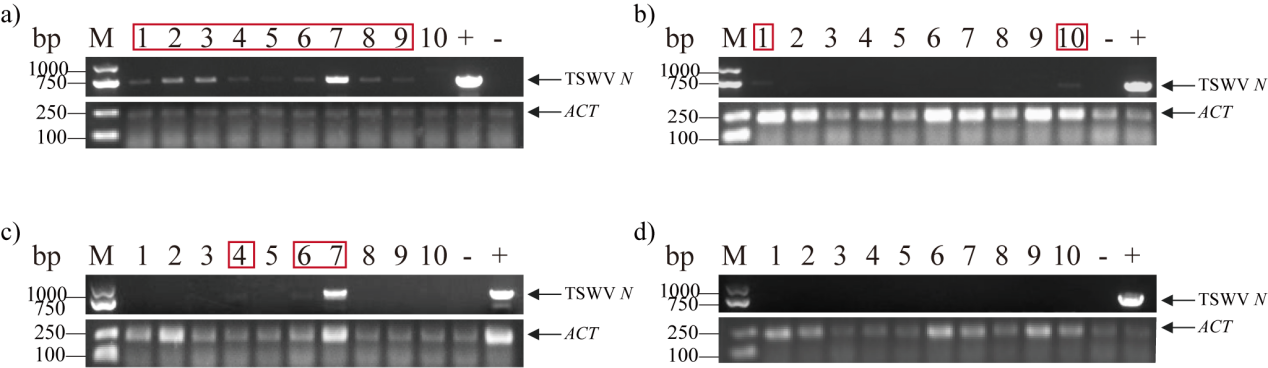


**Supplemental Fig 2** TSWV infection rate of progeny seedlings under different treatments. RT-PCR was performed to detect the TSWV *N* gene and *ACT* gene in progeny seedlings subjected to self-pollination of TSWV-infected pepper plants (a), pollination of virus-free mother plants with TSWV-infected pollen (b), pollination of TSWV-infected mother plants with virus-free pollen (c), and self-pollination of virus-free plants (d). M: DNA ladder; +, positive control; −, negative control. *ACT* was used as an internal reference gene. Red boxes indicate samples in which TSWV was detected. The TSWV *N* gene (777 bp) and *ACT* gene (228 bp) were amplified.


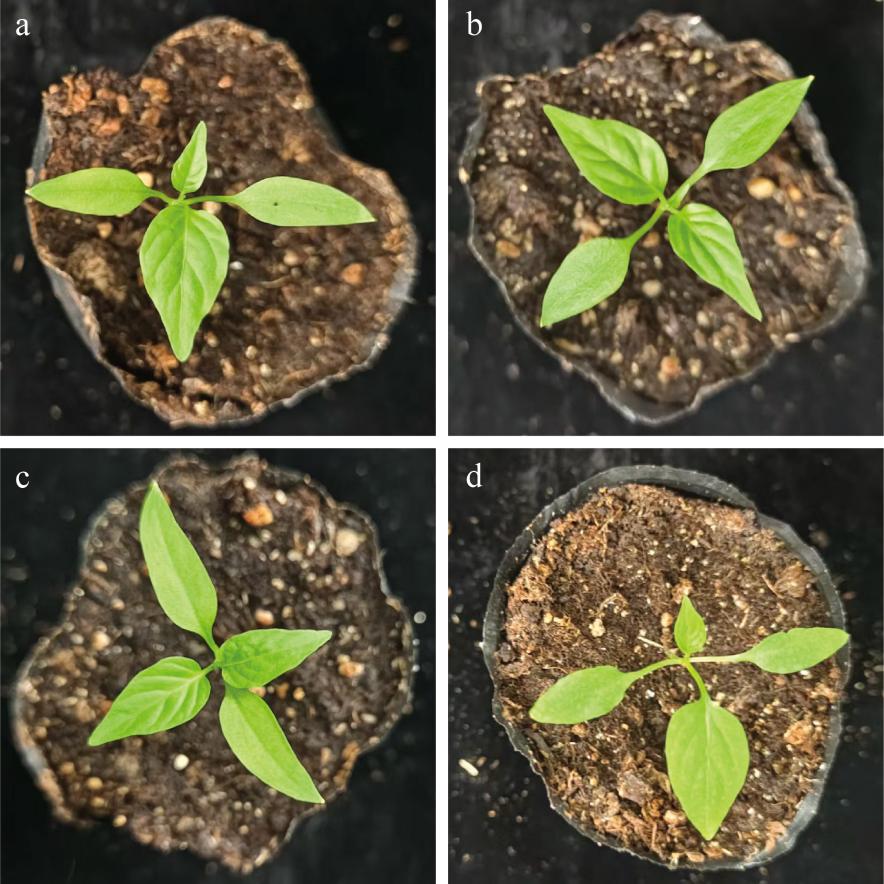


**Supplemental Fig 3** Symptoms of tomato spotted wilt orthotospovirus (TSWV) infected progeny seedling at different treatments. (a) self-pollination of TSWV-infected plants, (b) pollination of virus-free maternal plants with infected pollen, (c) pollination of infected maternal plants with virus-free pollen and (d) self-pollination of virus-free controls.
